# Ruhao Dashi granules exert therapeutic effects on H1N1 influenza virus infection by altering intestinal microflora composition

**DOI:** 10.3389/fmicb.2024.1482785

**Published:** 2024-10-09

**Authors:** Wei Pan, Rui Wu, Qianyun Zhang, Yuan Ma, Jinxiang Xiang, Jingbo Wang, Jing Chen

**Affiliations:** College of Life Science, Zhejiang Chinese Medical University, Hangzhou, China

**Keywords:** Ruhao Dashi granules, influenza, influenza A virus subtype H1N1, intestinal flora, Chinese medicine

## Abstract

**Objective:**

Antiviral medications for influenza could be ineffective due to the emergence of resistant influenza virus strains. Ruhao Dashi (RHDS) granules possess anti-inflammatory and antibacterial effects. The present study aimed to determine the efficacy of RHDS granules in treating influenza-infected mice and the mechanism underlying this treatment as well as its effect on the intestinal flora composition of the infected mice.

**Methods:**

The HPLC-UV method was used to identify the active components of RHDS granules. ICR mice were infected with influenza A virus (IAV) H1N1 subtype through a nasal drip. After the influenza mice model was successfully established, the pathological changes in the lungs were observed for 5 days after gavage treatment with 0.9% sterile saline and low, medium, and high doses (0.07, 0.14, and 0.28 g/mL, respectively) of RHDS granules. The serum levels of the cytokines IL-6 and TNF-*α* and sIgA were detected by ELISA. Real-time fluorescence quantitative PCR and western blotting assay were performed to determine the expression levels of the tight junction (TJ) proteins claudin-1, occludin, and zonula occludens-1 (ZO-1) in colon tissues. Furthermore, 16S rRNA gene sequencing of feces samples was conducted to assess the effect of RHDS granules on the gut microbiota.

**Results:**

RHDS granules exerted a protective effect on the lung tissues of IAV-infected mice; moreover, the granules reduced the synthesis of proinflammatory cytokines and increased the relative expression levels of claudin-1, occludin, and ZO-1 in colon tissues. Furthermore, RHDS granule treatment increased the relative abundance of *Lactobacillus*, *Akkermansia*, and *Faecalibaculum* and decreased the relative abundance of Muribaculaceae; thus, RHDS granules could stabilize the intestinal microbiota to some extent.

**Conclusion:**

RHDS granules exert a therapeutic effect on IAV-infected mice probably by modifying the structural composition of their intestinal microbiota.

## 1 Introduction

Influenza is a communicable respiratory tract infection caused by airborne influenza viruses and frequently occurs between the seasons of fall and early spring. The influenza virus causes mild to severe symptoms and is highly contagious (Keilman, 2019). Similar to acute febrile illness, influenza manifests as a range of systemic and respiratory symptoms such as runny nose, cough, chills, sore throat, malaise, and headache (Clohisey and Baillie, 2019). Influenza viruses evolve mainly through two mechanisms: antigenic transfer and antigenic drift. Co-infection of the same cells in an individual by two genetically distinct influenza virus strains results in swapping of their genomic segments. This recombination may lead to changes in the antigenic characteristics of the surface glycoproteins neuraminidase (NA) and hemagglutinin (HA); this phenomenon is referred to antigenic transfer (Kumari et al., 2023). In contrast, antigenic drift is a protracted process caused by the accumulation of mutations in viral surface proteins (Chen et al., 2022). HA, the most prevalent glycoprotein on the surface of influenza viruses, constantly undergoes changes to escape herd immunity induced by vaccination and spontaneous infection (Wu and Wilson, 2020; Wu and Wilson, 2017). NA inhibitors such as zanamivir and oseltamivir are frequently used to treat influenza. Although antiviral medications are the cornerstone of clinical treatment for influenza, their efficacy may be reduced or they may be rendered ineffective following the emergence of drug-resistant influenza viruses (Holmes et al., 2021). Hence, the development of strong antiviral medications is crucial to prevent and treat both seasonal and pandemic influenza virus infections.

Each person has a unique gut microbiota, which makes it a complex ecological system (Altieri et al., 2023). The gut-lung axis, which implies a significant relationship between the gut microbiota and the lungs, has been reported by recent research (Zhang et al., 2020). Current studies on influenza virus medications have focused on developing new NA inhibitors and HA blockers. Some studies have shown that mice infected with the influenza virus exhibit substantial intestinal inflammation and damage of their gut barriers (Guo et al., 2024). Furthermore, previous studies have also shown that the dysregulation of the intestinal flora impairs the ability of mice to mount an effective immune response; moreover, the intestinal flora can mitigate the immunological damage sustained by mice after receiving antibiotics through the Toll-like receptor 7 signaling pathway (Ichinohe et al., 2011; Wu et al., 2013). Elevated levels of colonic MUC5AC and fecal lipid transport protein-2 following viral lung infections suggest low-grade intestinal inflammation (Groves et al., 2018). Therefore, the use of the enteropulmonary axis as an additional treatment approach for influenza could be a novel and potentially beneficial strategy.

Ruhaodashi (RHDS) granules contain *Moslae Herba*, *Amomum tsao-ko Crevost et Lemarié*, *Magnolia officinalis* Rehd. et Wils, *Lonicerae japonicae flos*, *Artemisia annua* L., and *Atractylodes Lancea* (Thunb.) DC (Patent No. 202010234178.8). RHDS granules effectively disperse heat, generate moisture, and extract toxins. In clinical practice, therapy with RHDS granules has been shown to exert positive anti-inflammatory and antibacterial effects (Yi et al., 2023). However, it remains unknown whether RHDS granules can treat influenza by altering the intestinal flora. Therefore, in the present study, by using a pneumonia mice model infected with influenza A virus (IAV) subtype H1N1, we investigated how RHDS granules treat influenza by affecting the ecology and composition of the gut microbiota.

## 2 Materials and methods

### 2.1 Materials

#### 2.1.1 Experimental animals and viruses

Fifty-six male ICR mice, weighing 18 ± 2 g, were purchased from Hangzhou Medical College, with a laboratory animal production license number SYXK (zhe) 2021–0001. The IAV strain A/PR/8/34 (H1N1) was donated by the Institute of Basic Medical Sciences and Oncology, Chinese Academy of Sciences, and its 50% tissue culture infectious dose (TCID_50_) is 10^–3.4^. To avoid damage of the virus activity, the strain was stored properly at −80°C. Before each experiment, the appropriate amount of sample was removed as required to avoid repeated freezing and thawing.

#### 2.1.2 Medicines and reagents

RHDS granules were prepared by Harvest Pharmaceuticals Limited as packets containing 8 g of granules per packet. The granules were mixed with double-distilled water at a mass concentration of 10 mg/mL to form a stock solution. Bacterial contamination was removed by filtering the stock solution through a 0.22-μm filter membrane, and the filtered solution was stored at 4°C for later use. The remaining herbs and reagents are shown in Table 1.

**Table 1:** Experimental reagents and drugs.

Reagent name	Provider	Product number
Chlorogenic acid	The China Academy of Food and Drug Testing and Certification.	No. 110753–202,119
3,5-O-dicaffeoylquinic acid	The China Academy of Food and Drug Testing and Certification.	No. 111782–201,807
4,5-di-O-caffeoylquinic acid	The China Academy of Food and Drug Testing and Certification.	No. 111894–201,102
Honokiol	The China Academy of Food and Drug Testing and Certification.	No. 110730–201,915
Magnolo	The China Academy of Food and Drug Testing and Certification.	No. 111924–201,806
Atractylodin	The China Academy of Food and Drug Testing and Certification.	No. 110729–202,015
Oseltamivir	Born at Hoffmann-La Roche, Basel, Switzerland.	M1301
Shuanghuanglian Oral Liquid	Henan Fusen Pharmaceutical Co.	21,042,411
TRIzol^®^ Plus RNA Purification Kit	Thermo Fisher	12,183–555
RNase-Free DNase Set	Qiagen	79,254
SuperScript™III First-Strand Synthesis SuperMix	Thermo Fisher	11,752–05
Power SYBR^®^ Green PCR Master Mix	Applied Biosystems	4,367,659
T-PER Tissue Protein Extraction Reagent	Thermo Pierce	78,510
Halt Protease and Phosphatase Inhibitor Cocktail (100X)	Thermo Pierce	78,445
ECL DualVue WB Marker	GE	RPN810
SuperSignal West Dura Extended Duration Substrate	Thermo Pierce	34,075
Occludin	CST	91,131
Claudin-1	Abcam	ab180158
ZO-1	Thermo Fisher	40–2,200
β-actin	Abcam	ab68477
Goat anti-Mouse IgG(H + L)Secondary antibody	Thermo Pierce	31,160
Goat anti-Rabbit IgG(H + L)Secondary antibody	Thermo Pierce	31,210
Mouse tumor necrosis factor-alpha (TNF-α) ELISA kit	Hangzhou Risda Biotechnology Co.	MB-2868A
Mouse secretory immunoglobulin A (sIgA) ELISA kit	Hangzhou Risda Biotechnology Co.	MB-3166A
Mouse interleukin 6 (IL-6) ELISA kit	Hangzhou Risda Biotechnology Co.	MB-2899A

#### 2.1.3 Equipment

The following equipment were used in this study: CFX384 multiplex qRT-PCR instrument (Bio-Rad, USA); low-temperature, high-speed freezing centrifuge (Eppendorf, Germany); ultraviolet spectrophotometer (Beckman, USA); protein electrophoresis and transfer system (Bio-Rad); 7890B GC system for gas chromatography (Agilent); 5977B MSD mass spectrometer (Agilent); QT-1 vortex tester (Shanghai Qite Analytical Instruments Co., Ltd.). UltiMate 3,000 high-performance liquid chromatography (HPLC) system (including quaternary pump, injector, column oven, and UV detector; Thermo Scientific); Eclipse C18 column (4.6 × 150 mm, 5 μm, Agilent); electronic balance ME204E/02 (Mettler Toledo Instruments [SHANGHAI]); and KQ5200DE CNC ultrasonic cleaner (Kunshan Ultrasonic Instrument Co., Ltd.).

### 2.2 Methods

#### 2.2.1 Particle liquid chromatography of RHDS granules

##### 2.2.1.1 Chromatographic conditions

The following chromatographic conditions were applied: column: Eclipse C18 column; mobile phase: 0.1% phosphoric acid-water (A)-acetonitrile (B); gradient elution: (0 ~ 10 min, 10% → 20% B; 10 ~ 35 min, 20% → 55% B; 35 ~ 50 min, 55% → 90% B; 50 ~ 60 min, 90% B; 60 ~ 61 min, 90% → 10% B; 61 ~ 70 min, 10% B); wavelength: 280 nm; column temperature: 25°C; flow rate: 0.7 mL/min; injection volume: 10 μL.

##### 2.2.1.2 Preparation of mixed reference solutions

Reference standards of chlorogenic acid, 3,5-*O*-dicaffeoylquinic acid, 4,5-di-*O*-caffeoylquinic acid, honokiol, magnolol, and atractylodin were weighed, and 75% methanol was added to each of them to dissolve and prepare mixed reference solutions with mass concentrations of 300, 600, 200, 600, 200, and 100 μg/mL, respectively.

##### 2.2.1.3 Preparation of test solution

Wet granules (0.75 g) containing *Elsholtzia* and *Artemisia* were weighed, and 25 mL of 75% methanol was added. After ultrasonic extraction for 1 h, the mixture was left to cool. The loss in the weight of the mixture was compensated with 75% methanol. The renewed filtrate was passed through a 0.22-μm microporous filtration membrane to obtain the test solution.

#### 2.2.2 Subgroups, modeling, and interventions

Mice were randomly assigned to the following treatment groups comprising 8 mice each: control; model; Tamiflu; Shuanghuanglian (SHL); and RHDS low, medium, and high dose (RHDS-L, RHDS-M, and RHDS-H, respectively). Except for the control group, mice in the other groups were anesthetized using a gas anesthesia machine with 20 mL isoflurane. The mice were challenged intranasally with 10 times the median lethal dose (MLD50) of IAV (Zhang et al., 2018). None of the infected mice died. The RHDS granule dose was 9.1fold that of the clinical dose used in humans (converted according to the average body surface area assuming a body mass of 70 kg) as determined by performing calculations based on the difference in body weight between humans and mice. The low, medium, and high doses of RHDS granules were set as 0.07, 0.14, and 0.28 g/mL, respectively. These doses were administered to the infected mice through gavage at 24 h after IAV infection. The treatment group received one gavage per day for 5 days. An equal amount of saline was administered to the control and model groups simultaneously. These treatment regimens were continuously administered throughout the experiment.

#### 2.2.3 General observation and changes in lung and splenic indices

Each day, the mice were monitored for signs of infection, food and water intake, hair color, and activity. Five days after the final treatment, the mice were sacrificed, and their lungs and spleen were collected; these were washed with phosphate-buffered saline, dried on filter paper, and weighed. The lung and splenic indices were calculated as follows:


Lung index=weight of the lung÷body weight×100%.

Splenic index=weight of the spleen÷body weight×100%.

#### 2.2.4 Pathological changes in lung tissues

Fresh lung tissues were fixed with 4% paraformaldehyde, dehydrated, paraffin-embedded, and cut into 4-μm-thick serial sections. The tissues were stained with hematoxylin and eosin (H&E), and the pathological changes in these tissues were observed under a light microscope.

#### 2.2.5 ELISA for quantifying serum levels of the cytokines IL-6 and TNF-*α* and sIgA

Mice blood samples were collected in endotoxin-and pyrogen-free tubes and allowed to clot for 30 min at room temperature. The samples were then centrifuged at 1,000 × *g* for 10 min, and the serum was carefully separated. Next, 50 μL of standard and diluted samples was added to each microtiter well of a pre-coated enzyme plate. Horseradish peroxidase (HRP)-conjugated detection antibodies were then added to each well, and the plate was incubated for 60 min at room temperature. Subsequently, the wells were washed, a chromogenic substrate was added, and the plate was incubated for 15 min in the dark. Finally, color development was terminated, and the absorbance was measured at 450 nm within 30 min following the addition of the termination solution.

#### 2.2.6 qRT-PCR for detecting the degree of change in viral titer in the lung tissue of mice infected with the IAV influenza virus

To obtain a homogenate of lung tissue, a part of the tissue was ground, and the mixture was then centrifuged at 4,000 × g for 15 min at 4°C. Using GAPDH as the internal reference, the RNA concentration was ascertained and then computed using the 2^-ΔΔCt^ technique by the directions of the Hifair^®^ II 1st Strand cDNA Synthesis Kit (see Table 2).

**Table 2:** Sequences of primers for viral titers.

Gene	Forward (5′-3′)	Reverse (5′-3′)
FluA	GACCRATCCTGTCACCTCTGAC	AGGGCATTYTGGACAAAKCGTCTA
GAPDH	GAAGGTCGGTGTGAACGGATTTG	CATGTAGACCATGTAGTTGAGGTCA

#### 2.2.7 qRT-PCR for determining the mRNA expression levels of the TJ proteins claudin-1, occludin, and zonula occludens-1 (ZO-1) in mucosal tissues

To obtain the colon homogenate, a portion of the colon tissue was grounded and centrifuged at 4,000 *g* for 15 min at 4°C. In accordance with the manufacturer’s instructions, total RNA was extracted using TRIzol reagent, reverse transcribed into cDNA, and subjected to PCR amplification (PCR conditions: 95°C for 2 min, 95°C for 15 s, 62°C for 30 s, 72°C for 30 s, ×40 cycles). Each sample was analyzed in triplicate. GAPDH was used as the internal reference gene, and target gene expression was calculated relative to GAPDH expression by using the 2^−ΔΔCt^ method. The primers are shown in Table 3.

**Table 3:** Primers for qRT-PCR.

Gene	Forward (5′-3′)	Reverse (5′-3′)
*Claudin 1*	CCTGCCCCAGTGGAAGATTTACT	GTGCTTTGCGAAACGCAGGACAT
*Occludin*	GCGATCATACCCAGAGTCTTTC	GGTGTCTCTAGGTTACCATTGC
*ZO-1*	CCATGACTCCTGACGGTTGGTCTT	CGGATCTCCAGGAAGACACTTGT
*GAPDH*	GAAGGTCGGTGTGAACGGATTTG	CATGTAGACCATGTAGTTGAGGTCA

#### 2.2.8 Western blotting assay for measuring the relative expression levels of claudin-1, occludin, and ZO-1 in mucosal tissues

A portion of mouse colon tissue was harvested and grounded by adding RIPA buffer. The grounded sample was centrifuged, and the supernatant was used as the total protein solution. The total protein extraction kit (containing a protease inhibitor cocktail) was used to extract the total protein. After measuring the protein concentration using the BCA kit, equal amounts of proteins (25 μg) were taken and separated by 10% sodium dodecyl sulfate-polyacrylamide gel electrophoresis. The separated proteins were then transferred to a polyvinylidene fluoride membrane. The membrane was blocked with 5% skimmed milk for 1 h at room temperature. Subsequently, the membrane was incubated with primary antibodies against occludin (1:1,000), claudin-1 (1:2,000), ZO-1 (1:500), and *β*-actin (1:10,000) at 4°C overnight.

The membrane was then incubated with HRP-conjugated sheep anti-rabbit and sheep anti-mouse IgG secondary antibodies (1:5,000) for 1 h at room temperature. Subsequently, the enhanced chemiluminescence kit was used for color development, and the gray value of each band was measured by Image J software. The expression level of each target protein was calculated relative to that of β-actin as the internal reference protein.

#### 2.2.9 High-throughput sequencing of 16S rRNA gene sequences in enteric bacteria

The colonic feces of each group were collected and stored in a refrigerator at −80°C. The 16S rRNA V3 ~ V4 regions were sequenced by Illumina MiSeq sequencing technology at Shanghai Parsonage Biotechnology Co. Alpha diversity analysis (Chao1 index, Shannon index, Simpson index, and Observed_species index), beta diversity analysis (distance matrix with principal coordinate analysis [PCoA] and non-metric multidimensional scaling [NMDS] analysis), and other analytical methods were used to obtain relevant information regarding changes in the gut microbiota composition.

### 2.3 Statistical analysis

GraphPad Prism 8.0.1 and SPSS 22.0 were used for statistical analysis. The group comparisons were operated by one-way analysis of variance (ANOVA) and *T*-test. *p*-value <0.05 was considered to be statistically significant.

## 3 Results

### 3.1 Particle liquid chromatography of RHDS granules

**Figure 1:**
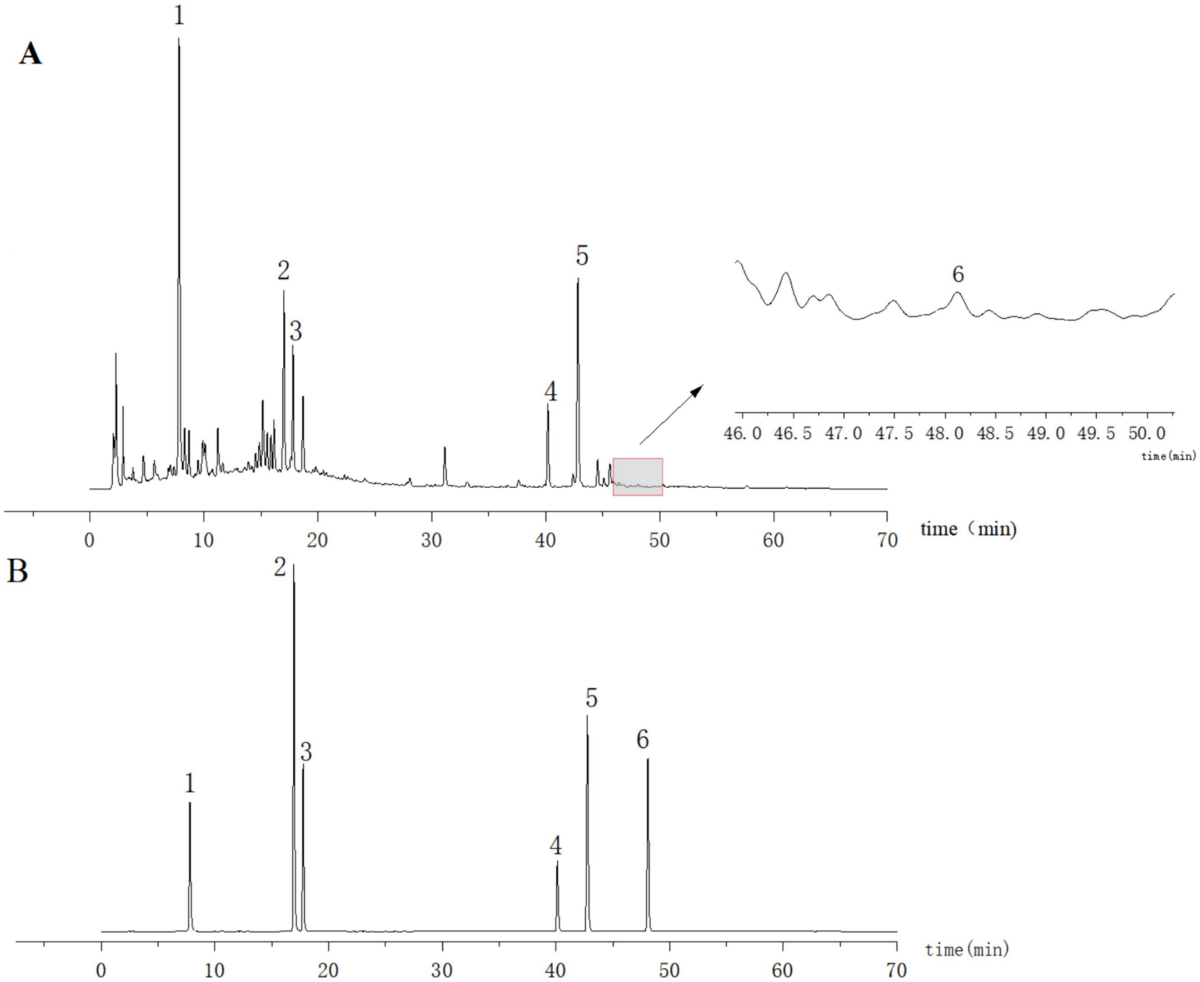
HPLC plots of the test solution **(A)** and mixed control solution **(B)**. 1: Chlorogenic acid; 2: 3,5-*O*-Dicaffeoylquinic acid; 3: 4,5-Di-*O*-caffeoylquinic acid; 4: Honokiol; 5: Magnolol; 6: Atractylodin.

Ten microliters of each of the control and test solutions were taken and injected into the sample, and the chromatogram was then recorded. The results showed that the retention time of the six components in the test solution was consistent with that of the control (Figure 1). The separation from the adjacent peaks was >1.0 under the condition, and the theoretical plate number was not less than 17,000 according to chlorogenic acid.

### 3.2 Effects of RHDS granules on the general health state of mice and lung indices

**Figure 2:**
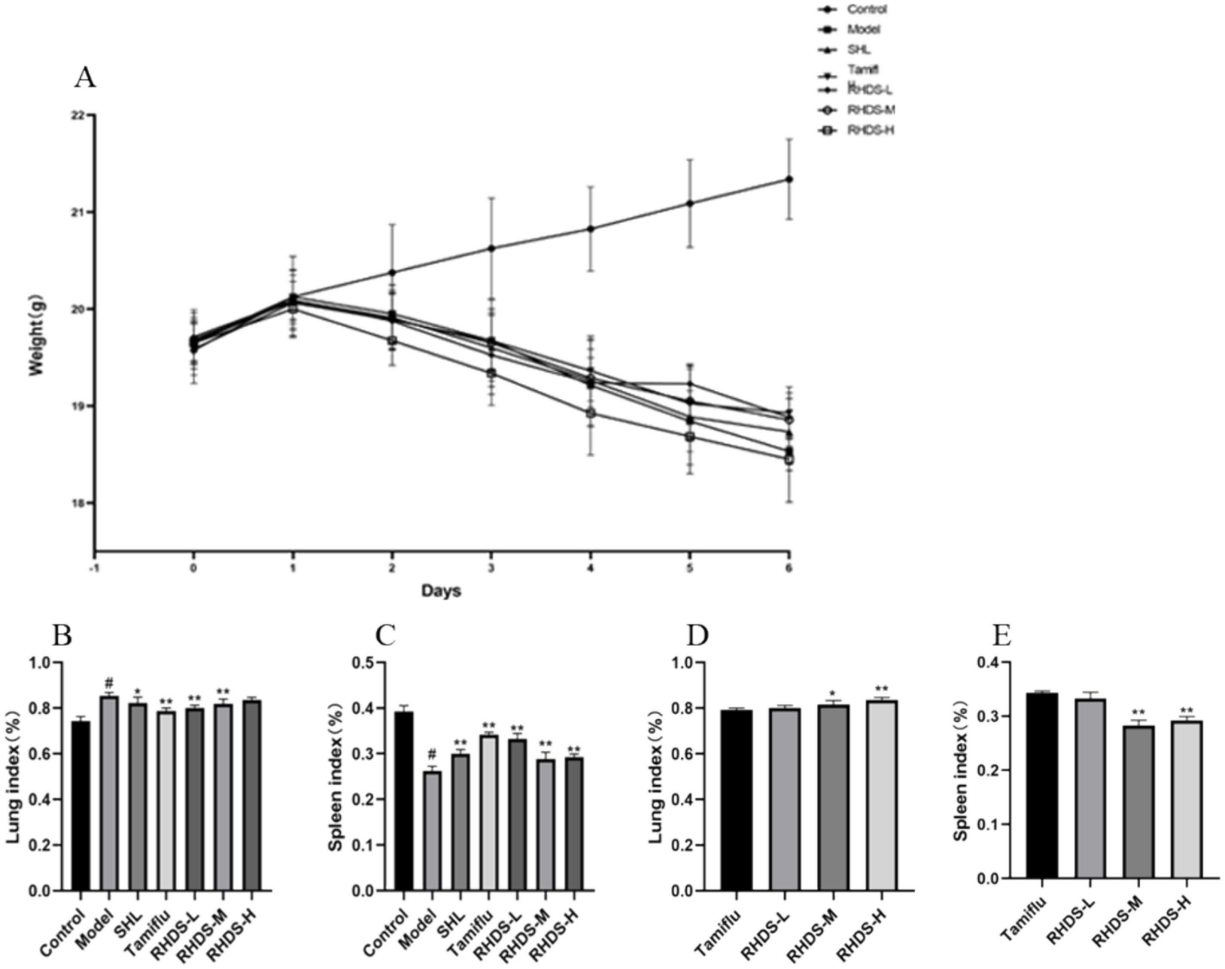
Changes in body weight and the lung and splenic indices after influenza infection in mice. Following IAV infection, mice lost body weight and showed apparent influenza symptoms **(A)**. Mice with a significant increase in the lung index **(B)**. Mice with a significant decrease in the splenic index **(C)**. The results of the study showed that the effect of the RHDS low-dose treatment group was closest to that of the Tamiflu treatment group **(D,E)**.^#^*p* < 0.05 vs. control group, ^*^p < 0.05 vs. model group, ^**^p < 0.01 vs. model group, ^*^p < 0.05 vs. Tamiflu group, ^**^p < 0.01 vs. Tamiflu group.

Mice in the control group had shiny coats, were energetic and mentally healthy, and had gained weight naturally. Within 1–2 days of IAV infection, mice in the infection group showed classic influenza symptoms, such as dull fur, arched backs, curled bodies, shortness of breath, and steady weight loss. Compared to the model group, the Tamiflu, RHDS-L, and RHDS-M groups exhibited a significant decrease in the symptoms of arched backs, shortness of breath, and dull fur. The differences in the body weight of mice in each group are shown in Figure 2A. Compared to the control group, the model group showed a significant increase in the lung index (*p* < 0.05) and a significant decrease in the splenic index (*p* < 0.05). Furthermore, compared to the model group, the SHL, Tamiflu, RHDS-L, and RHDS-M groups showed a significant decrease in the lung index (*p* < 0.05, *p* < 0.01, *p* < 0.01, and *p* < 0.01, respectively) and a significant increase in the splenic index (*p* < 0.01). As can be seen from the graph, RHDS with Tamiflu works better. Therefore, in this study, the Tamiflu and RHDS treatment groups were also statistically analyzed. The results of this study concluded that the RHDS low-dose group was closest to the Tamiflu group. (Figures 2B,C). Statistical analyses were conducted on the Tamiflu treatment group and the RHDS treatment group. The results of the study showed that the effect of the RHDS low-dose treatment group was closest to that of the Tamiflu treatment group.The results are shown in (Figures 2D,E).

### 3.3 Effects of RHDS granules on histopathological changes in mice lungs

**Figure 3:**
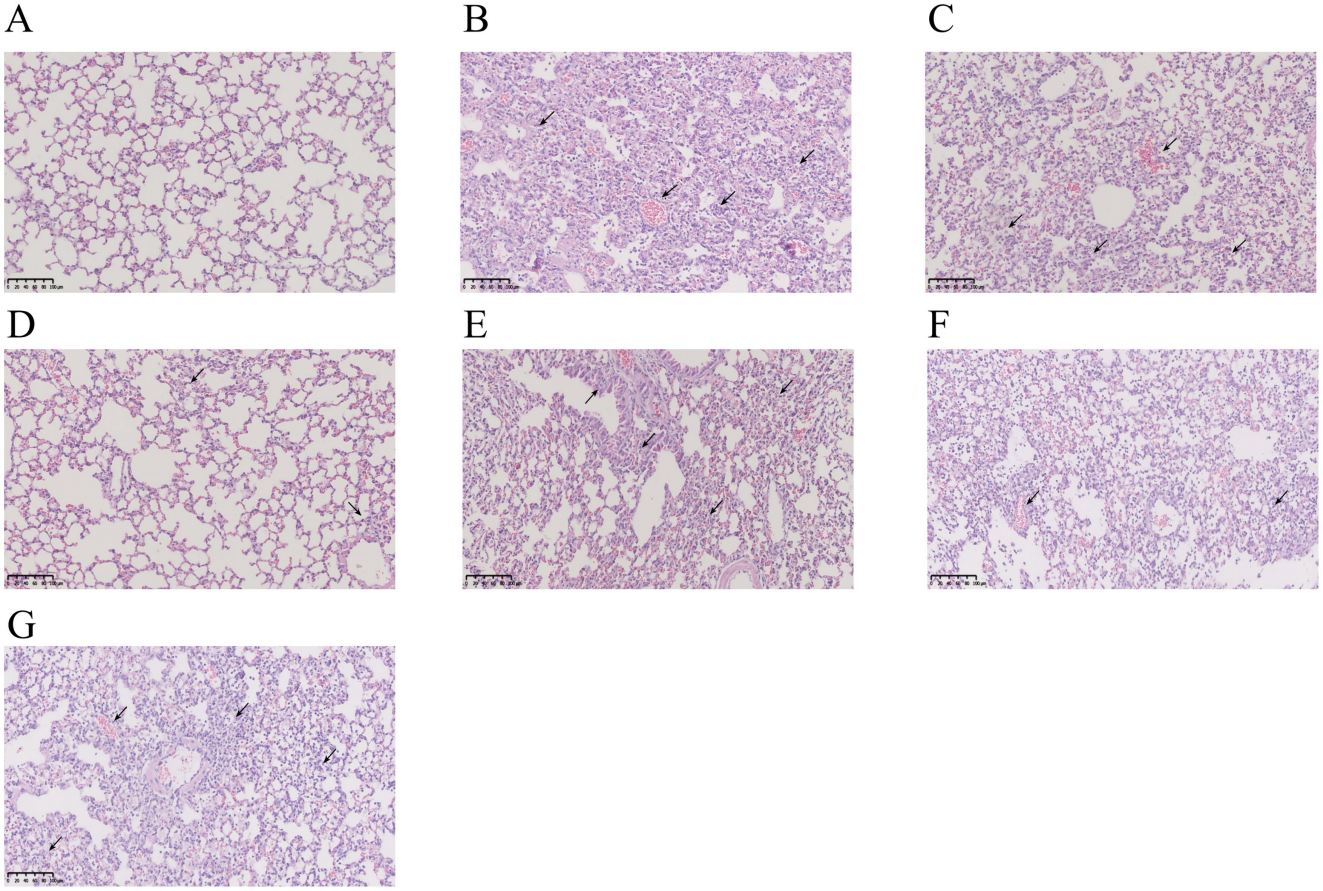
Photographs of pathological changes in the lungs of mice infected with IAV for 5 days. The lungs of the control group were structurally intact with no inflammatory cell infiltration **(A)**. The model group showed a large number of inflammatory cell infiltration and alveolar congestion in the lungs **(B)**. The SHL group had focal areas of solid lesions infiltrated with inflammatory cells in the lungs **(C)**. The Tamiflu group showed infiltration of a small number of inflammatory cells in the lungs **(D)**. Inflammatory cell infiltration in the lung tissues of all RHDS groups was ameliorated to varying degrees **(E–G)**.

As shown by H&E staining, the lung tissues of mice in the control group had a clean and intact alveolar structure, with thin alveolar walls, no inflammatory secretions in the alveolar lumen, and no infiltration of inflammatory cells. Compared to the control group mice, the model group mice exhibited a greater quantity of inflammatory cells in the alveolar lumen, together with thickening of the alveolar septum and hemorrhage. All mice groups treated with RHDS showed a reduction in the inflammatory infiltration in lung tissues. However, localized infiltration of inflammatory cells was still noted, along with alveolar wall weakening, minimal alveolar wall congestion, and no appreciable thickening of the alveolar septa. Although the Tamiflu group showed fewer alveolar wall septa, some alveoli were still infiltrated with a small number of inflammatory cells. In the SHL group, focal areas of solid lesions were infiltrated with inflammatory cells, and solid areas with structurally intact alveoli were absent. This finding suggests that RHDS has a therapeutic effect on the lungs of mice with influenza (see Figure 3).

### 3.4 Effect of RHDS granules on the serum levels of IL-6, TNF-*α*, and sIgA

**Figure 4:**
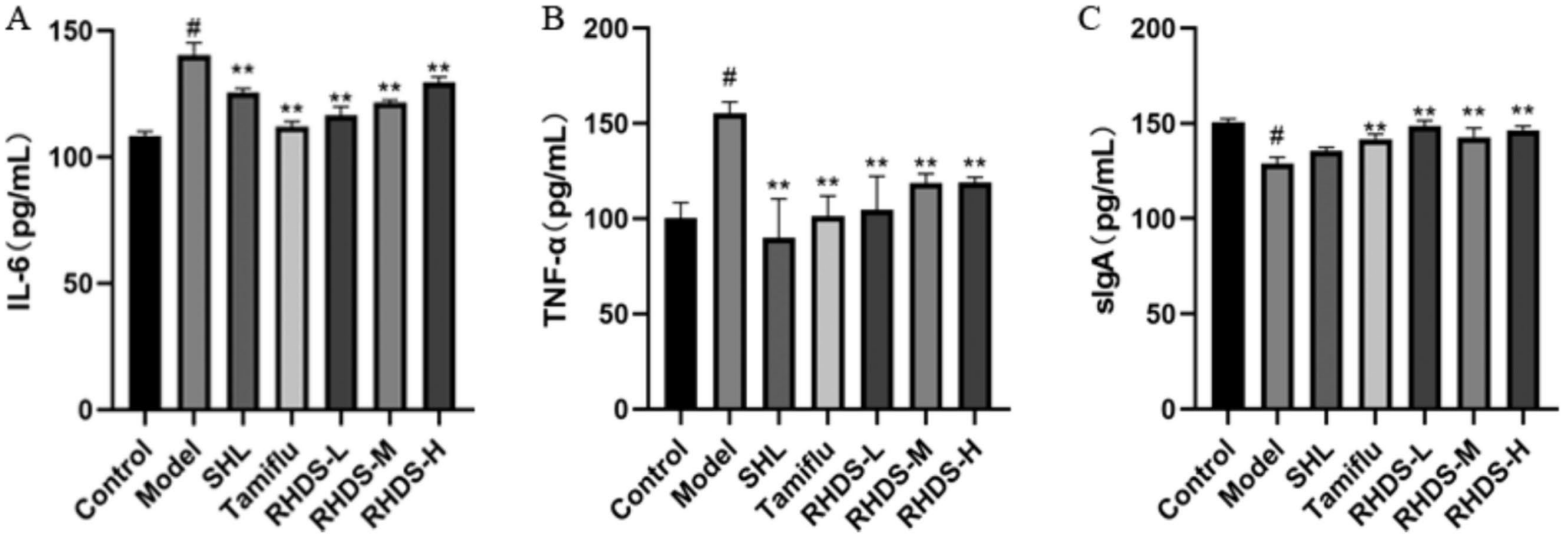
Serum levels of IL-6, TNF-*α*, and sIgA as determined by ELISA **(A–C)**. The ELISA results showed significantly higher levels of TNF-α and IL-6 and significantly lower levels of sIgA in the model group as compared to those in the control group. The Tamiflu and RHDS-L groups exhibited significantly lower levels of IL-6 and TNF-α and significantly higher levels of sIgA. ^#^*p* < 0.05 vs. control group, ^*^p < 0.05 vs. model group, ^**^p < 0.01 vs. model group.

The ELISA results showed a significant increase in IL-6 and TNF-α levels and a significant decrease in sIgA levels in the model group as compared to those in the control group (*p* < 0.05). Furthermore, compared to the control group, the SHL, Tamiflu, and RHDS groups showed a significant reduction in IL-6 levels (*p* < 0.01). The slgA levels were significantly increased in the Tamiflu and RHDS groups (*p* < 0.01). Moreover, the TNF-α levels were highly significantly reduced in the SHL, Tamiflu, and RHDS-L groups (*p* < 0.01) and significantly decreased in the RHDS-M and RHDS-H groups (*p* < 0.05) (see Figure 4).

### 3.5 Variations in viral titers determined via RHDS in the lung tissue of IAV influenza virus-infected mice

**Figure 5:**
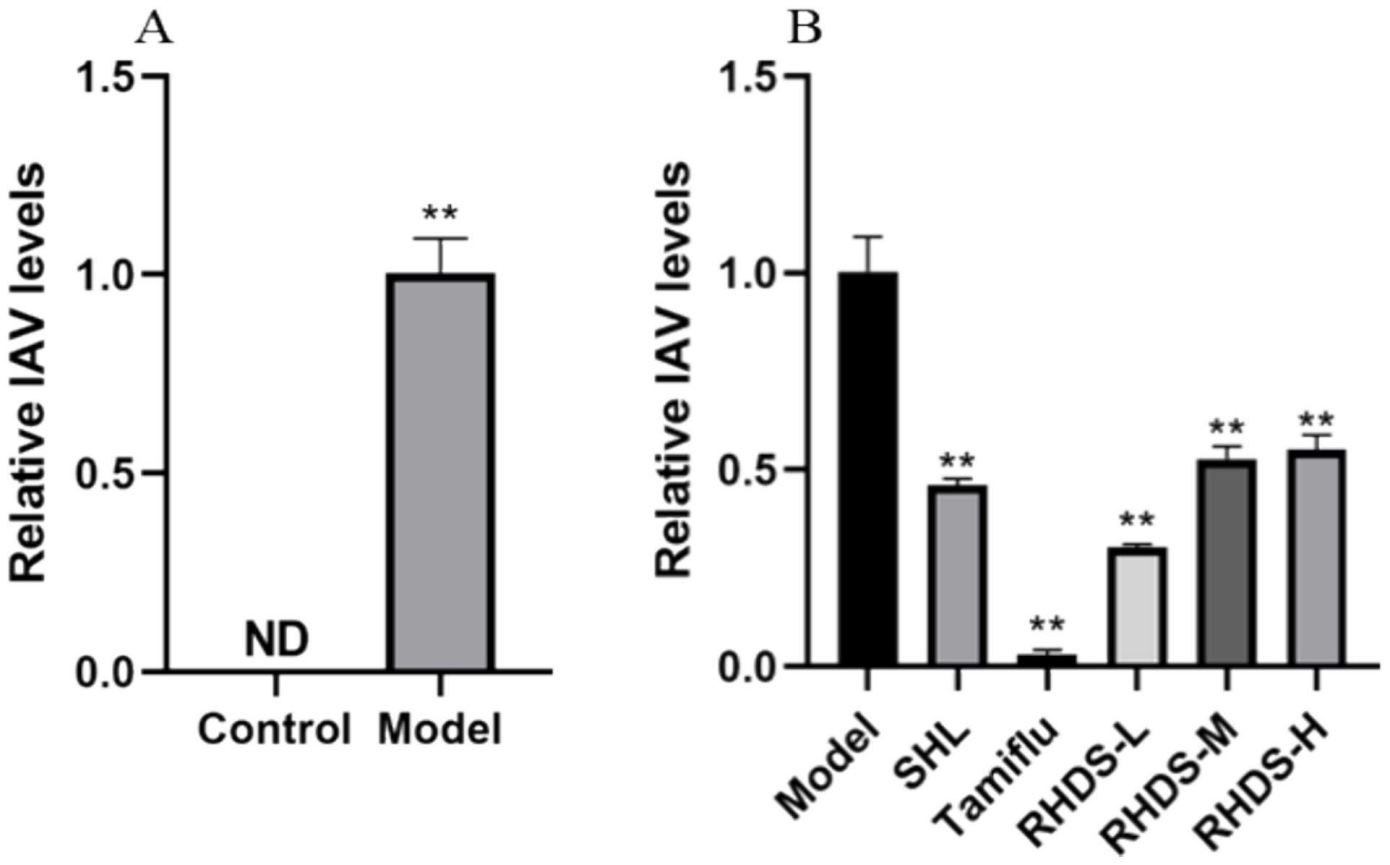
Variation in the viral titer level in IAV mouse lung tissues. IAV viral titers in the lung tissue of the model group of mice were significantly elevated, while RHDS in all dose groups and the positive treatment group’s lung tissue had significantly lower levels of viral titers., ^**^p < 0.01.

According to the qRT-PCR results, the control mice did not exhibit any viral detection. The model group’s mice’s lung tissues had a considerably greater viral titer of IAV (*p* < 0.01) than the animals in the control group (Figure 5A). The low-dosage group of RHDS had the greatest effect among all the dose groups, and the results are displayed in Figure 5. Compared with the model group, the viral titer reduced dramatically in both the positive drug group and the administered group (Figure 5B).

### 3.6 Effects of RHDS granules on the mRNA and protein expression levels of claudin-1, occludin, and ZO-1

Based on the qRT-PCR results, the model group showed significantly lower mRNA expression levels of claudin-1, occludin, and ZO-1 in their colon tissues as compared to the control group (*p* < 0.05). The mRNA expression levels of these proteins were significantly higher in the colon tissues of the SHL, Tamiflu, and RHDS-L group than in the colon tissues of the model group (*p* < 0.01). The RHDS-M group showed a significant increase in the mRNA expression levels of occludin and ZO-1 (*p* < 0.01).

**Figure 6:**
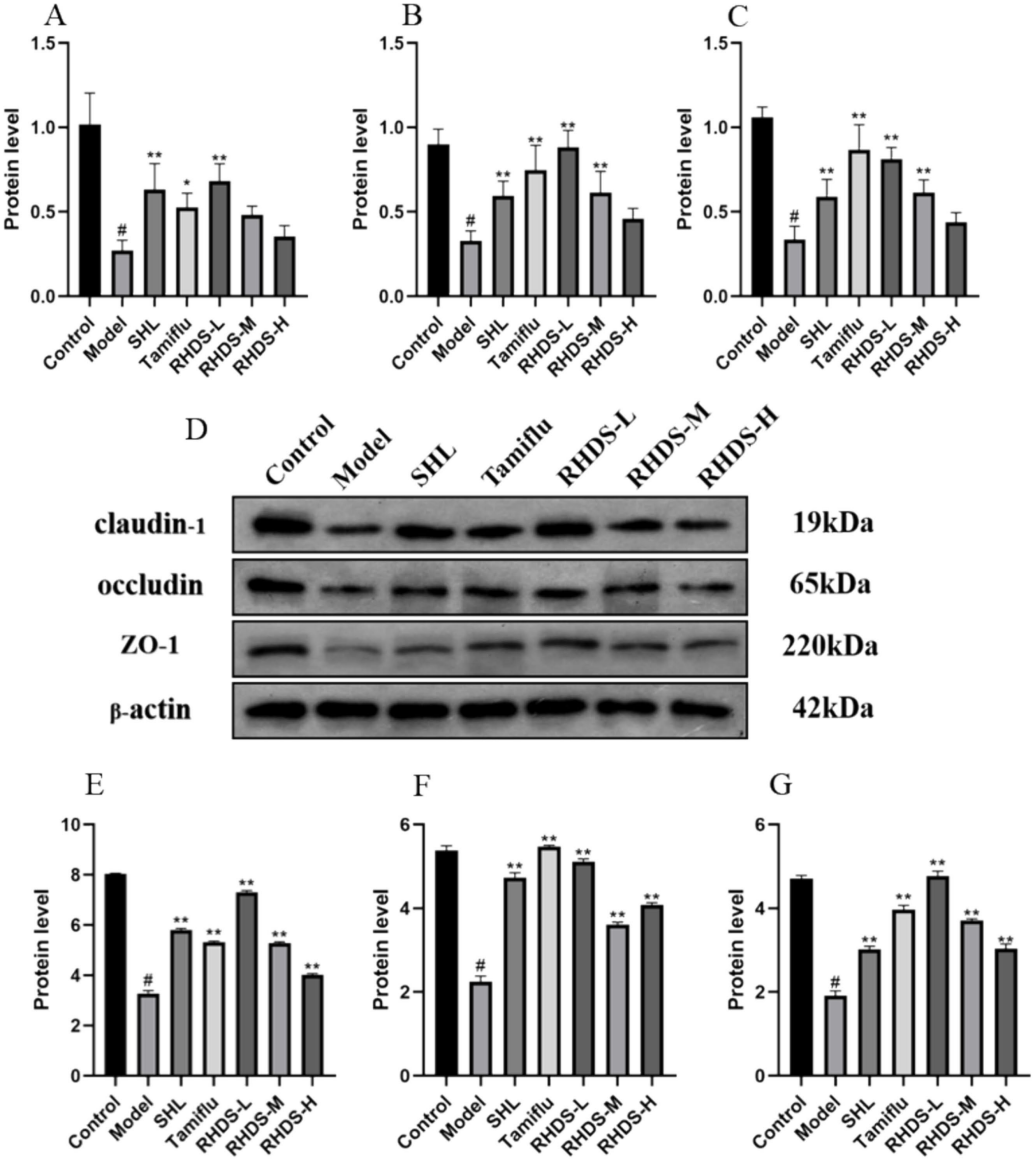
The mRNA expression levels of claudin-1, occludin, and ZO-1 were measured by qRT-PCR in the colon tissues **(A–C)**. Protein expression levels of claudin-1, occludin, and ZO-1 were measured by western blotting assay in the colon **(D–G)**. **(D)** Shows an image of the bands in the western blotting assay from top to bottom for claudin-1, occludin, ZO-1, and the internal reference. The mRNA and protein expression levels of claudin-1, occludin, and ZO-1 were significantly lower in the model group than in the control group, while they were significantly higher in the RHDS treatment groups. ^#^*p* < 0.05 vs. control group, ^**^p < 0.01 vs. model group.

Western blotting assay revealed lower protein expression levels of claudin-1, occludin, and ZO-1 in the colon tissues of model group mice than in the colon tissues of control group mice (*p* < 0.05). Furthermore, the protein expression levels of claudin-1, occludin, and ZO-1 were significantly higher in the colon tissues of mice in the RHDS, SHL, and Tamiflu groups than in the colon tissues of model group mice (*p* < 0.01) (Figure 6).

### 3.7 Analysis of the intestinal microflora composition

**Figure 7:**
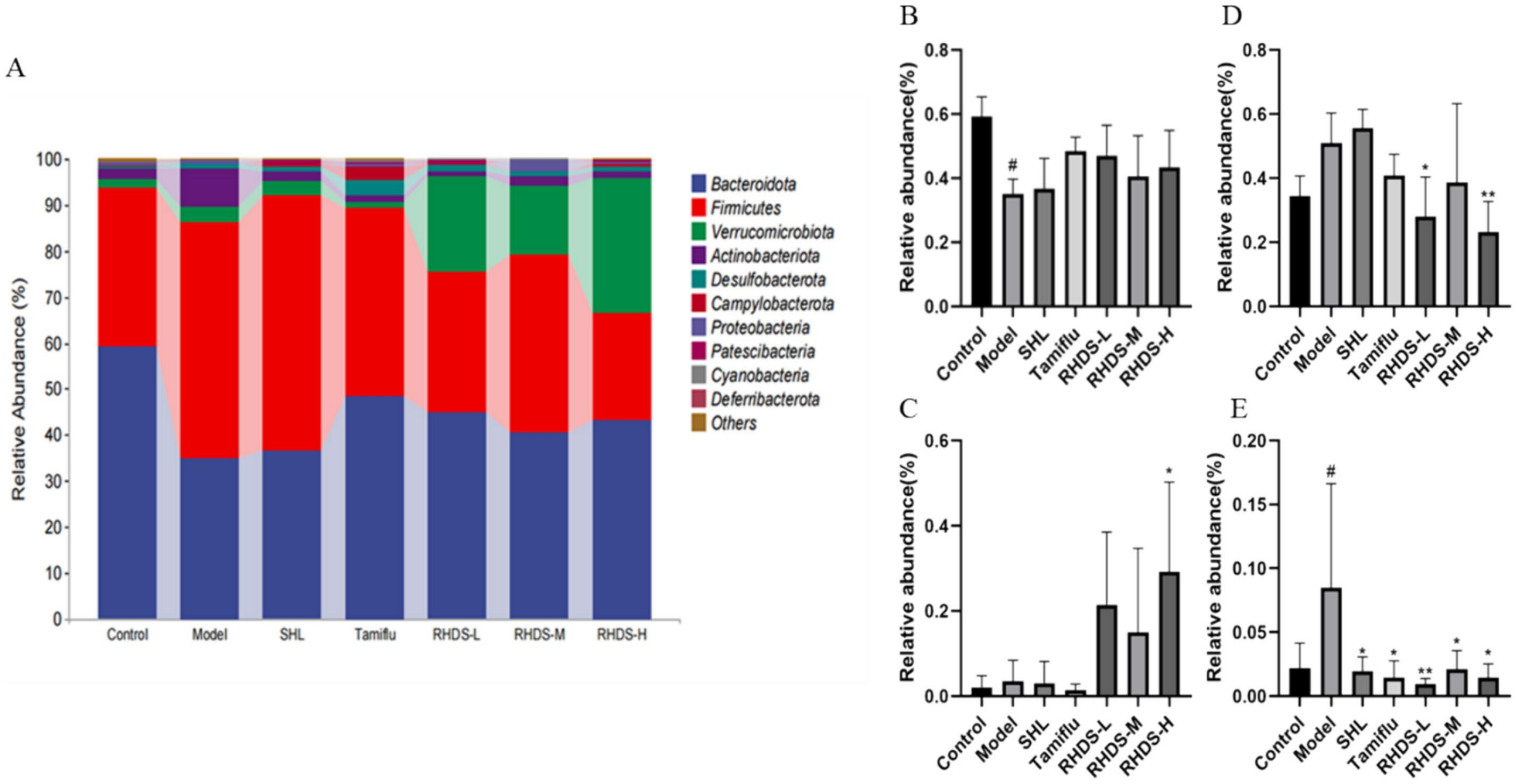
The composition of the intestinal microflora of mice was analyzed at the phylum level **(A)**. **(B–E)** Represent Bacteroidetes, Verrucomicrobiota, Firmicutes, and Actinobacteriota, respectively. Compared to the model group, the RHDS-L and RHDS-H groups showed a significant decrease in the relative abundance of Firmicutes, and the RHDS-H group exhibited a significant increase in the relative abundance of Verrucomicrobiota. The relative abundance of Actinobacteriota was significantly decreased in the SHL, Tamiflu, RHDS-M, and RHDS-H groups. ^#^*p* < 0.05 vs. control group, ^**^p < 0.01 vs. model group.

**Figure 8:**
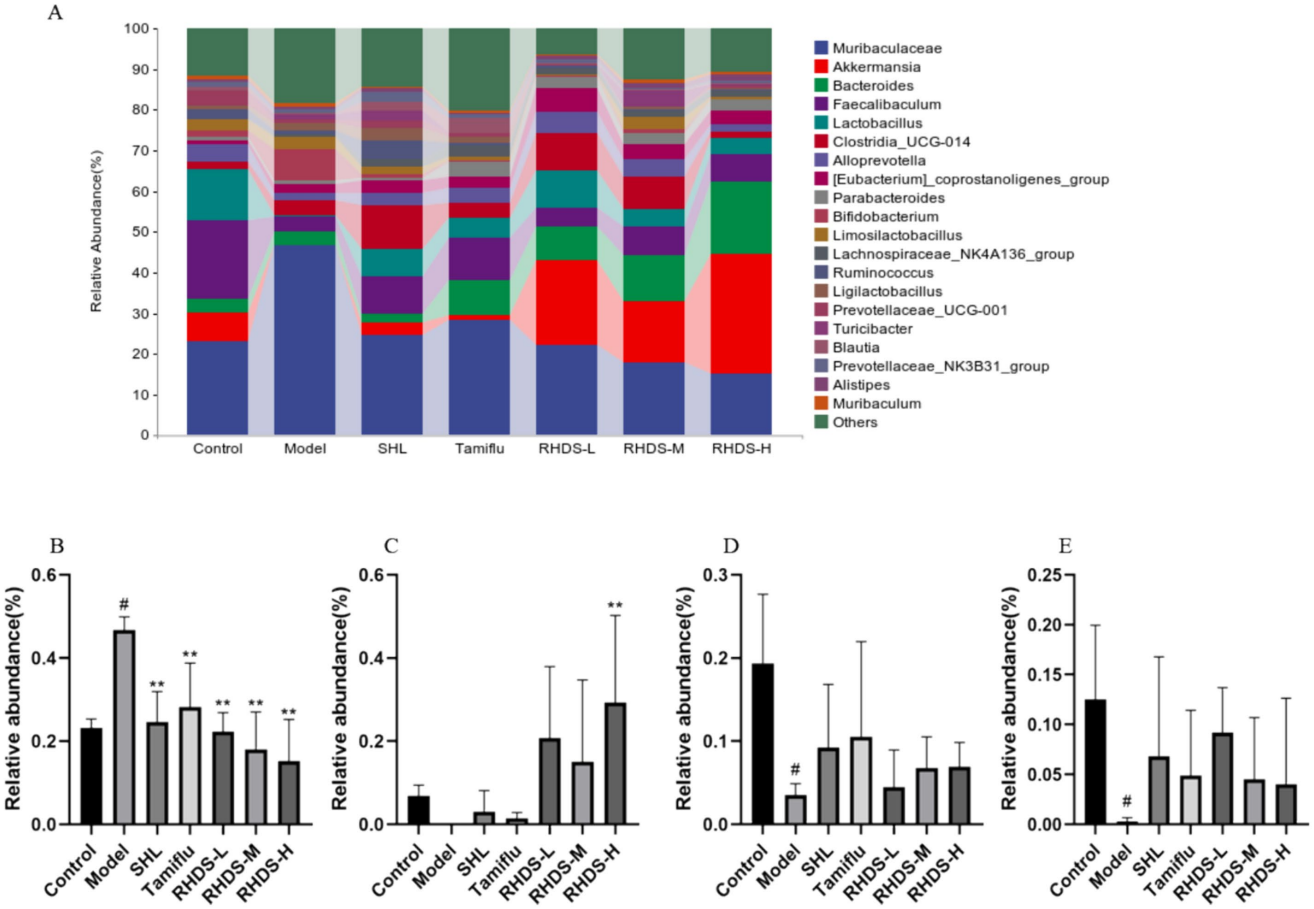
Analysis of the mice colonic microflora at the genus level **(A)**. Compared to the control group, the model group showed a significant increase in the relative abundance of Muribaculaceae **(B)** and a significant decrease in the relative abundance of Faecalibaculum **(D)** and Akkermansia **(E)**. The relative abundance of Lactobacillus **(C)** increased significantly and that of Muribaculaceae decreased significantly in the RHDS-H group as compared to those in the model group. ^#^*p* < 0.05 vs. control group, ^**^p < 0.01 vs. model group.

The structural composition of bacterial colonies isolated from mouse colonic fecal samples was analyzed at the phylum and genus levels by using QIIME software. Figure 7A shows the categorical bar clustering of species composition for each group at the phylum level. The mouse intestinal microbiota comprised 10 phyla, including Bacteroidetes, Firmicutes, Verrucomicrobiota, Actinobacteriota, Desulfobacterota, Campylobacterota, Proteobacteria, Patescibacteria, Cyanobacteria, and Desferriobacteria. Of these, the phyla Bacteroidetes and Firmicutes showed the highest abundance. The relative abundance of Firmicutes and Verrucomicrobiota tended to increase in the model group as compared to that in the control group. Furthermore, compared to the model group, the RHDS-L and RHDS-H groups showed significantly lower relative abundance of Firmicutes (*p* < 0.05 and *p* < 0.01, respectively), and the RHDS-H group exhibited significantly higher relative abundance of Verrucomicrobiota (*p* < 0.05). The relative abundance of Actinobacteriota was significantly higher in the model group than in the control group (*p* < 0.05). Compared to the model group, the relative abundance of the SHL group, Tamiflu group, RHDS-M group, and RHDS-H group were significantly decreased (*p* < 0.05). The relative abundance of the RHDS-L group was highly significantly decreased (*p* < 0.01) The results are shown in (Figures 7B–E). Figure 8A shows the top 20 genera for each group. The relative abundance of Muribaculaceae was significantly higher, while that of Faecalibaculum and Lactobacillus was significantly lower (*p* < 0.05) in the model group as compared to those in the control group. Furthermore, compared to the model group, the SHL, Tamiflu, and RHDS groups exhibited significantly lower relative abundance of Muribaculaceae (*p* < 0.01), the RHDS-H group showed significantly higher relative abundance of Akkermansia (*p* < 0.01), and all the RHDS groups displayed an increasing trend in the relative abundance of Faecalibaculum and Lactobacillus. The results are shown in Figures 8B–E.

### 3.8 Analysis of the intestinal microflora composition and differential species analysis at the phylum and genus levels

**Figure 9:**
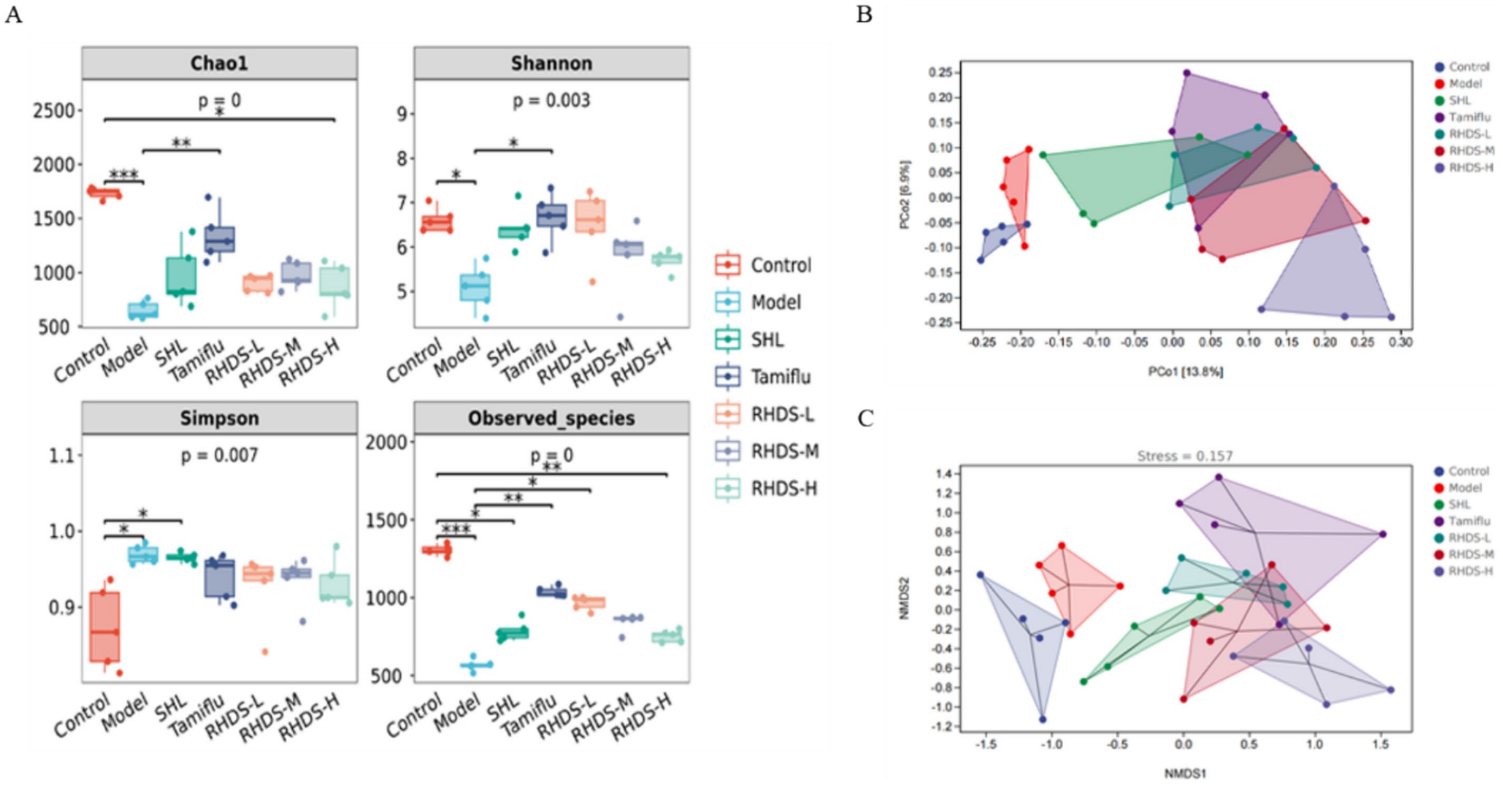
Alpha diversity and beta diversity analyses of the mouse intestinal flora. Alpha diversity analyses included the results for Chao1 index, Shannon index, Simpson index, and Observed_species index **(A)**. Compared to the control group, the model group showed significantly lower Chao1, Shannon, and Observed_species indices and significantly higher Simpson index. Furthermore, compared to the model group, the Tamiflu group showed significantly higher Chao1, Shannon, and Observed_species indices, and the RHDS-L group showed significantly higher Observed_species index. ^**^P / ^***^p < 0.01 vs. control group, ^*^ *P/* ^**^p < 0.01 vs. model group. Beta diversity analyses **(B,C)** showed that the control group was clearly separated from the model group, the RHDS-M group tended to move closer to the control group along the PCo2 axis, and RHDS granule treatment reduced the variation in the composition of the intestinal microflora between IAV-infected mice and control mice.

Alpha diversity and beta diversity indices are used to characterize the diversity of microbial species within and between habitats, respectively, to comprehensively evaluate their overall diversity. Alpha diversity is mainly reflected by the Chao1 index, Shannon index, Simpson index, and Observed_species index. As shown in Figure 9A, Chao1, Shannon, and Observed_species indices were significantly lower in the model group than in the control group (*p* < 0.01, *p* < 0.05, and *p* < 0.01, respectively), and the Simpson index was significantly higher (*p* < 0.01). Compared to the model group, the Tamiflu group showed significantly higher Chao1 index, Shannon index, and Observed_species index (*p* < 0.01, *p* < 0.05, and *p* < 0.01, respectively), and the RHDS-L group showed significantly higher Observed_species index (*p* < 0.05). These results showed that the abundance and diversity of the intestinal microflora were significantly reduced in IAV mice, and RHDS improved the imbalance of the intestinal microflora and increased its diversity in mice with influenza.

The samples were also analyzed by distance matrix with PCoA and NMDS analysis, and the results are shown in Figures 9B,C. According to the PCoA results, the control group was separated from the model group along the PCo2 axis, and the RHDS-M group tended to converge toward the control group along the PCo2 axis. The stress value was 0.125 in the NMDS analysis. This finding suggests a partial difference in the gut microbial composition between the control and model groups; furthermore, RHDS could reduce variability between the gut flora of IAV-infected mice and control mice.

## 4 Discussion

Influenza is an acute respiratory illness caused by influenza viruses. It poses a substantial risk to human health as it can develop into a severe illness in susceptible individuals (Li and Cao, 2017). NA inhibitors and influenza vaccination are the two primary treatment approaches used to treat influenza patients. The development of vaccines for influenza is challenging and constrained because influenza viruses are constantly evolving through mutations; thus, novel anti-influenza medications need to be developed despite the potential side effects and drug resistance of NA inhibitors. In a healthy intestinal environment, the intestinal flora maintains a dynamic balance. However, the defenses of the intestinal flora are compromised under certain conditions that interfere with this balance; this increases intestinal permeability, leading to immunological diseases of the intestinal tissues (Wedgwood et al., 2020). Intricate bidirectional lymphatic and blood exchange between the lungs and the gut creates an intrapulmonary axis that interacts with factors responsible for illness development (Bingula et al., 2017). Increased amounts of pro-inflammatory cytokines in the bloodstream are thought to modify the gut microbiota and jeopardize the intestinal epithelium’s integrity. As a result, the patient develops a “leaky gut, “which allows intestinal bacterial products and antigens to enter the bloodstream and exacerbate the illness (Sun et al., 2022). Bacterial metabolites and debris can enter the bloodstream and travel to distant organs when the intestinal barrier’s permeability is compromised, potentially affecting immune cells (Damiani et al., 2023). Thus, it is anticipated that a thorough investigation of the composition and activities of the gut microbiota will play a significant role in the search for novel therapeutic approaches for respiratory disorders (Hong, 2023). This indicates that targeting the lung-gut axis could open up a new approach to preventing and treating influenza. Therefore, the current study proposed that RHDS could potentially alleviate influenza through the use of the gut microbiota.

In this study, the nasal drip model was used to infect mice with IAV. The results demonstrated that IAV-infected mice showed depression, emaciation, and weight loss. Several cytokines, including interferons, proinflammatory factors, and chemokines, are produced following the penetration of the mucosal barrier by the influenza virus, adsorption of the virus on the cells, and its invasion of the respiratory epithelial cells (Sato and Kiyono, 2012). In the present study, the results for the pulmonary splenic index, pathological changes in treated and untreated mice, and ELISA demonstrated that the IL-6 and TNF-*α* levels were significantly increased in IAV-infected mice and significantly decreased in RHDS granule-treated mice. These findings suggest that RHDS could inhibit the production of proinflammatory cytokines, thereby alleviating lung inflammation and reducing the immunological damage caused by influenza viruses.

The TJ, which is formed by interactions between claudin, ZO-1, and members of the tight junction-associated marvel protein family, is a protein complex that plays a role in the first line of mucosal defense, i.e., the epithelial barrier (Kuo et al., 2022). The structure and function of the TJ largely depend on the claudin family of proteins, and higher expression of claudin-1 in this area suggests higher expression of inflammatory markers (Pope et al., 2014; Weber et al., 2008). Pathogens or antigenic components of pathogens cannot penetrate the gut’s lamina propria while the mucosal barrier is intact, and both humans and animals require a balanced intestinal microbiological environment to preserve homeostasis (Wang et al., 2022). The results of the present study indicate that RHDS treatment can mitigate the damage caused by the influenza virus to colonic tissues and exert a protective effect on the mucosal barrier. Specifically, the expression levels of claudin-1, occludin, and ZO-1 were significantly decreased in the colonic tissues of IAV-infected mice and increased to varying degrees in the RHDS dose groups. The present study also found that abnormalities in the composition of intestinal microbiota were mostly manifested in the model group in terms of increase in the abundance of harmful bacteria such as Muribaculaceae and a decrease in the abundance of beneficial bacteria such as *Lactobacillus*, *Faecalibaculum*, and *Akkermansia*. Probiotic use is a preventive or therapeutic strategy for autoimmune and chronic inflammatory diseases, as certain probiotic bacteria in the gut can alleviate these conditions (Green et al., 2020). *Akkermansia muciniphila* produces acetate and propionate; furthermore, a recent study revealed that Amuc_2109, an enzyme secreted by *A. muciniphila*, attenuates dextran sodium sulfate-induced colitis, increases TJ protein expression, and decreases NLRP3 inflammatory vesicle expression in mice (Rodrigues et al., 2022; Qian et al., 2022). *Faecalibaculum rodentium*, a recently identified antitumor strain, can inhibit the growth of tumor cells and activate calmodulin phosphatase by reducing NFATc3 during the initial stages of tumor development. Thus, this strain could be a possible biomarker for the early detection of tumors (Zagato et al., 2020). Probiotic strains belonging to the Lactobacillus and Bifidobacterium genera are the most frequently reported to offer protection against influenza infection (Groeger et al., 2020). These probiotic strains protect mice from influenza infection and have anti-inflammatory properties (Kato et al., 2023).

Treatment with *Lactobacillus* can enhance animal health, alleviate clinical symptoms, lower lung viral load, and increase patient survival (Lehtoranta et al., 2014). A previous study reported that oral treatment with *Lactobacillus* YYC-3 suspension suppressed the NF-κB signaling pathway and downregulated the expression levels of inflammatory factors (Yue et al., 2020).

## 5 Conclusion

The present study found that treatment with RHDS granules can minimize the immunological damage caused by influenza viruses to the body, prevent the emergence of IAV-induced lung inflammation, and suppress the production of proinflammatory cytokines. These effects could be accomplished by modifying the composition of the gut microbiota. Thus, this study indicates that RHDS granules have anti-influenza virus properties and that one of the mechanisms underlying these antiviral effects may be through the regulation of the intestinal microbiota composition by RHDS granules.

## Data Availability

The datasets presented in this study can be found in online repositories. The name of the repository and accession number can be found below: National Center for Biotechnology Information (NCBI)BioProject, https://www.ncbi.nlm.nih.gov/bioproject/, PRJNA1166069.
